# In Silico Studies Reveal Peramivir and Zanamivir as an Optimal Drug Treatment Even If H7N9 Avian Type Influenza Virus Acquires Further Resistance

**DOI:** 10.3390/molecules27185920

**Published:** 2022-09-12

**Authors:** Edita Sarukhanyan, Tipack Ayothyapattanam Shanmugam, Thomas Dandekar

**Affiliations:** Department of Bioinformatics, Biocenter, Am Hubland, University of Würzburg, 97074 Würzburg, Germany

**Keywords:** H7N9 influenza virus, neuraminidase, mutation, binding pocket, molecular docking

## Abstract

An epidemic of avian type H7N9 influenza virus, which took place in China in 2013, was enhanced by a naturally occurring R294K mutation resistant against Oseltamivir at the catalytic site of the neuraminidase. To cope with such drug-resistant neuraminidase mutations, we applied the molecular docking technique to evaluate the fitness of the available drugs such as Oseltamivir, Zanamivir, Peramivir, Laninamivir, L-Arginine and Benserazide hydrochloride concerning the N9 enzyme with single (R294K, R119K, R372K), double (R119_294K, R119_372K, R294_372K) and triple (R119_294_372K) mutations in the pocket. We found that the drugs Peramivir and Zanamivir score best amongst the studied compounds, demonstrating their high binding potential towards the pockets with the considered mutations. Despite the fact that mutations changed the shape of the pocket and reduced the binding strength for all drugs, Peramivir was the only drug that formed interactions with the key residues at positions 119, 294 and 372 in the pocket of the triple N9 mutant, while Zanamivir demonstrated the lowest RMSD value (0.7 Å) with respect to the reference structure.

## 1. Introduction

H7N9 influenza A virus became a serious threat to human health after a first outbreak in February 2013. The next epidemic wave occurred in June 2014, which caused 450 human infections, including 165 fatalities [1,2].

Influenza A virus has two surface glycoproteins—hemagglutinin (HA) and neuraminidase (NA)—which are divided into subtypes. There are 18 different HA subtypes and 11 different NA subtypes [1,2]. For instance, H7N9 type virus has an HA of H7 subtype and an NA of N9 subtype [1,3]. HA binds the sialic acid-containing receptor on the surface of the host cell and promotes the fusion of the virus [1,4]. NA, instead, cleaves the bond between the sialic acid of host cell receptor and the newly formed virions, thus facilitating the release of the last ones [1,5]. The freshly released viruses represent an infection threat for healthy cells. Therefore, NA plays a key role in virus spread in the human body. Without NA, the influenza A virus would be limited to only one replication cycle, which is not sufficient for serious infection [1,6]. In other words, NA is a key enzyme and represents an attractive target for anti-influenza drugs. The overall dynamics of all influenza A virus mutations are complex and trigger from time to time major outbreaks and even a pandemic. Therefore, we focus here on H7N9 influenza A virus and its neuraminidase and try to study major drug-resistant mutations before they occur.

Several targets and the corresponding drugs have already been suggested for inhibition of the influenza virus replication cycle. For instance, the drugs amantadine and rimantadine are known as M2-ion channel blockers [7]. Favipiravir (T-705) is effective for the viral RNA polymerase inhibition [7]. Ribavirin, known as a nucleoside inhibitor, mainly targeting viral RNA-dependent RNA polymerase inhibition, has also demonstrated high antiviral activity to both wild type and mutated H7N9 virus [8]. Similarly, the drug Alferon N (IFN-alpha) was suggested to be highly effective against the above-mentioned types of H7N9 virus [9].

The most frequently used drugs for influenza virus treatment, approved by the World Health Organization, are Oseltamivir, Zanamivir, Peramivir and Laninamivir [1,10,11]. These drugs are known to target the N9 type of NA and inhibit its activity. However, the virus can easily mutate and acquire resistance towards known medications. To date, there is already evidence for the R to K mutation in N9 at position 294 (A/Shanghai/1/2013), which caused severe resistance to the drug Oseltamivir [12]. However, resistance was not that strong towards Zanamivir, Peramivir and Laninamivir. Several drugs have been suggested to improve binding to the active site of the mutated subtype of NA. For example, Liu et al. [13] in their work suggested quercetin, chlorogenic acid, oleanolic acid and baicalein—the bioactive components from traditional Chinese plant medicine. According to the obtained results, these compounds have shown stronger bindings to the active site of R294K mutant NA rather than Oseltamivir. Other possible inhibitors showing better binding properties were suggested by the group of He et al. [1], where they show stronger binding of L-Arginine, Aspartame, D-Arginine and Benserazide hydrochloride towards the active site of mutated N9 neuraminidase compared to Oseltamivir carboxylate.

According to the data provided by Wu et al. [12], the three arginines at positions 372, 119 and 294 in the catalytic site of N9 enzyme play a key role in the glycosidic bond cleavage between the newly formed virus and the host cell. For this reason, we focused our attention on these three particular arginines. The evidence of mutation in one of these key residues, in particular R294K, already exists [1,12]. Our assumptions concerning the next possible mutations refer to the other two arginines at positions 119 and 372, in particular, their single, double and triple mutations to lysine. According to the Blosum default matrix Blosum62 [14], substitution of arginine to lysine is highly favorable. Therefore, to prevent an epidemic of the virus with such kinds of mutations and avoid an anticipated drug resistance in advance, in the current research work, besides the wild type and R294K mutation, we also focused our attention on possible R119K, R372K, R119_294K, R119_372K, R372_294K and R119_294_372K mutations in N9 subtype of NA. In order to evaluate the best drug candidate, we tested, against N9 with such mutations, the existing drugs, known for their strong anti-influenza properties (Oseltamivir, Zanamivir, Peramivir, Laninamivir, L-Arginine, Benserazide hydrochloride) [1,12] using different molecular docking algorithms.

## 2. Results

### 2.1. Validation of Docking

**Figure 1:**
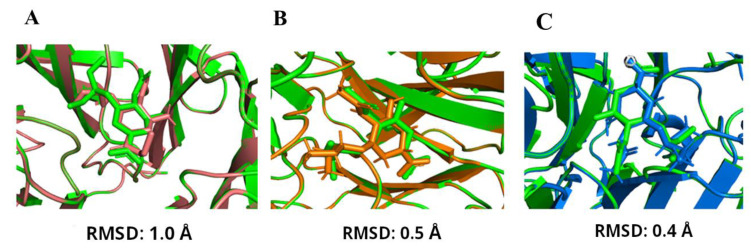
(**A**) Superposition of the drug Oseltamivir inside the binding pocket of native crystal structure of 4MWQ shown in green and the re-docked complex using “AutoDock” is shown in deep salmon (RMSD: 1.0). (**B**) Superposition of the drug Oseltamivir inside the binding pocket of native crystal structure of 4MWQ shown in green and the re-docked complex using “GOLD” is shown in orange (RMSD: 0.5). (**C**) Superposition of the drug Oseltamivir inside the binding pocket of the native crystal structure of 4MWQ shown in green and the re-docked complex using “MOE” is shown in blue (RMSD: 0.4).

Prior to estimation of the best drug candidate against native as well as mutated NAs, we validated the docking software. Figure 1 shows the superposition of Oseltamivir carboxylate structures within the crystal structure of NA (4MWQ) with the docked one. The superposition resulted in a positional root-mean square deviation (RMSD) value of 1.0 Å performed with AutoDock software (Figure 1A), 0.5 Å with GOLD software (Figure 1B) and 0.4 Å with MOE (Figure 1C). The values are within the admissible error range (well below 2 Å) and therefore the docking results can be considered as trustworthy.

### 2.2. Evaluation of The Best Drug Candidates against Wild Type As Well As Mutated N9

Table 1, Table 2 and Table 3 report the top scores for the drugs Oseltamivir, Zanamivir, Peramivir, Laninamivir, L-Arginine and Benserazide hydrochloride according to three docking protocols—AutoDock, GOLD and MOE—with respect to the binding pocket of the wild type as well as mutated N9 NAs. From all studied compounds with respect to all considered NAs, the highest score in most of the cases is found with the drug Peramivir according to the three docking algorithms (please refer to Table 1, Table 2 and Table 3). Despite the fact that Peramivir maintains the best binding score in almost all studied NAs, it is worth noting that mutation in the binding pocket reduces the strength of binding (Table 1, Table 2 and Table 3, top scores highlighted in bold).

### 2.3. Binding Assessment of Potent Drugs with H7N9 Single, Double and Triple Mutants

Table 4 and Table 5 report the list of the residues inside the pockets of NAs involved in hydrogen bond (H-bond) and salt bridge formations, respectively, with the drugs Oseltamivir, Zanamivir, Peramivir, Laninamivir, L-Arginine and Benserazide hydrochloride.

According to the H-bond data from Table 4, the most frequently observed pocket residues involved in H-bond formations are Arg 153, Arg/Lys 372, Arg/Lys 294 and Arg/Lys 119 for the drug Oseltamivir; Arg/Lys 119, Trp 180, Arg/Lys 372, Asp 152, Tyr 406 and Arg/Lys 294 for Zanamivir; Trp 180, Arg 153, Asp 152, Tyr 406, Arg/Lys 294 and Arg/Lys 372 for Peramivir; Arg/Lys 294, Arg/Lys 372, Glu 278, Arg 153, Trp 180 and Tyr 406 for Laninamivir; Trp 180, Asp 152 and Tyr 406 for L-Arginine; and, finally, Trp 180, Glu 229, Tyr 406, Asp 152 and Arg/Lys 294 for Benserazide hydrochloride.

It is worth noting that the residues Arg/Lys 294 and Tyr 406 can be considered as the key residues since they are involved in H-bond formation for almost all drug–enzyme complexes.

The average number of H-bonds in the existence of NA inhibitors ranges from 6 for Zanamivir and Laninamivir; 5 for Peramivir and Benserazide hydrochloride; 4 for L-Arginine; 5 for Benserazide hydrochloride; and 3 for Oseltamivir. As for the average number of residues involved in salt bridge formations with the drugs, it is about 5 for Peramivir and L-Arginine, 4 for Zanamivir and Laninamivir, and 0 for Benserazide hydrochloride. Taken together, the binding profile in terms of H-bond formation and salt bridge interaction looks quite similar for the drugs Zanamivir, Peramivir and Laninamivir.

### 2.4. Influence of Mutations on Drug Fitness to the Binding Pocket of NAs

**Figure 2:**
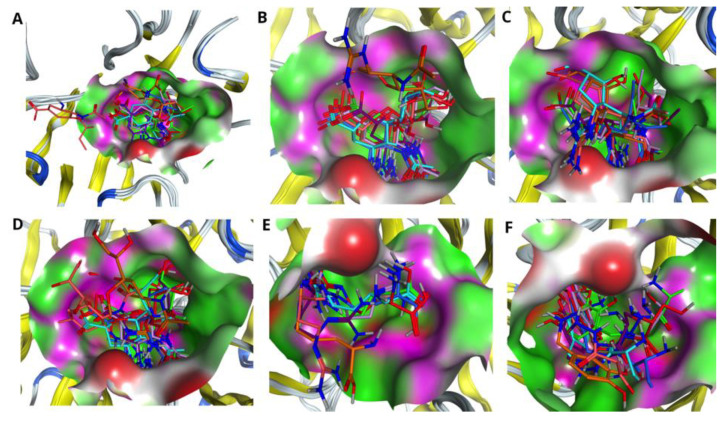
Comparing drug fit to wild type and mutated NA binding pockets. Superposition of (**A**) Oseltamivirs, (**B**) Zanamivirs, (**C**) Peramivirs, (**D**) Laninamivirs, (**E**) L-Arginines and (**F**) Benserazide hydrochlorides in the pockets of native NA (4MWQ) (red) as well as NAs with single (R294K) (cornflower blue), R119K (green), R372K (purple), double R294_119K (orange), R294_372K (turquoise), R119_372K (pink) and triple R294_119_372K (salmon) mutations.

It is effective to look at the impact of mutations in the H7N9 NA catalytic site, as it leads to conformational changes of the binding pocket as well as various poses of drugs inside the cavity. Figure 2 demonstrates the superposition between Oseltamivirs (A), Zanamivirs (B), Peramivirs (C), Laninamivirs (D), L-Arginines (E) and Benserazide hydrochlorides (F) taken from docking of the drugs with respect to the pocket of wild type (used here as a reference) as well as mutated N9 NAs. It is worth noting that the most affected by R to K mutation inside the active site are the drugs Oseltamivir, L-Arginine and Benserazide hydrochloride. The calculated values of RMSDs for such drugs show a huge shift with respect to the structure bound to the wild type N9 (please refer to Table 6). Mutation of the pocket residues had less of an impact on Zanamivir, Peramivir and Laninamivir. As Table 6 shows, single mutations (R294K, R119K and R372K) do not affect significantly the binding poses of the drugs; the values of RMSD lie within 1.5 Å for the drugs Zanamivir and Laninamivir and are less than 1 Å for the drug Peramivir. The drastic conformational changes as well as shift of the binding pose in all drugs causes the double R294_119K mutation inside the binding pocket of N9 (see Figure 2A–F, all drugs are highlighted in orange, and Table 6). In this case the most affected drug is Zanamivir with an RMSD value of 7.3 Å; the least affected is the drug Peramivir with an RMSD value of 4.6 Å. Triple R294_119_372K mutation also caused significant displacement of the binding poses in all considered drugs, except Zanamivir (see Figure 2A–F, all drugs are highlighted in salmon, and Table 6). In this case, the most affected by such mutations is the drug Oseltamivir with an RMSD value of 10.8 Å and the least affected is Zanamivir with an RMSD value of 0.7 Å.

### 2.5. Identification of the Best Fitting Drug to the Pocket

**Figure 3:**
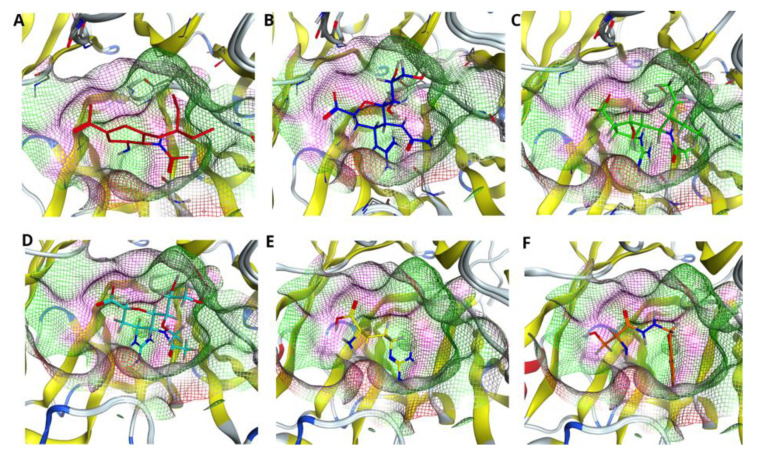
Comparing drug fit to wild type NA binding pocket. Accommodation of the drugs (**A**) Oseltamivir (red), (**B**) Zanamivir (blue), (**C**) Peramivir (green), (**D**) Laninamivir (cyan), (**E**) L-Arginine (yellow) and (**F**) Benserazide hydrochloride (orange) in the pocket of native H7N9 neuraminidase, with reference to the pdb code 4MWQ.

To identify the best fitting drug to the H7N9 pocket, we evaluated each drug accommodation inside the pocket of wild type NA as well as one with single (R372K), double (R294_119K) and triple (R294_119_372K) mutations. Figure 3 illustrates the fitness of the drugs Oseltamivir (A), Zanamivir (B), Peramivir (C), Laninamivir (D), L-Arginine (E) and Benserazide hydrochloride (F) to the pocket of wild type N9 NA. As Figure shows, all drugs are positioned in the cavity of the pocket. However, proper filling of the pocket is observed only for the drugs Zanamivir, Peramivir and Laninamivir. Oseltamivir, L-Arginine and Benserazide hydrochloride do not fill the pocket completely, probably due to the fact that they score less than the above-mentioned drugs (see Table 1, Table 2 and Table 3, R294 column).

**Figure 4:**
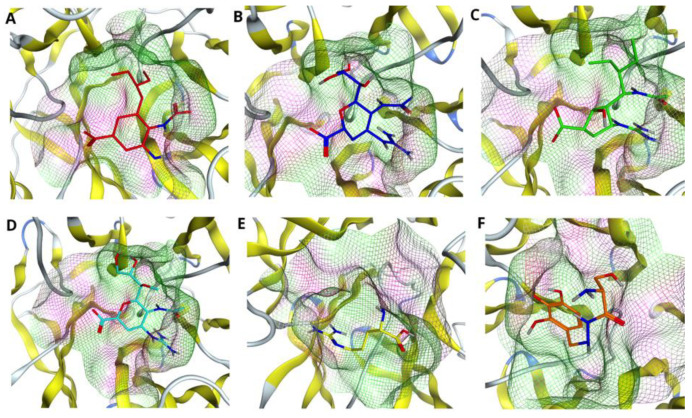
Comparing drug fit to single mutated NA binding pocket. Accommodation of the drugs (**A**) Oseltamivir (red), (**B**) Zanamivir (blue), (**C**) Peramivir (green), (**D**) Laninamivir (cyan), (**E**) L-Arginine (yellow) and (**F**) Benserazide hydrochloride (orange) in the pocket of H7N9 neuraminidase with single R372K mutation.

Figure 4 demonstrates the drugs inside the pocket of N9 with single R372K mutation. As the figure shows, the mutation influenced the shape of the pocket; however, it did not affect significantly the position of the ligand inside the pocket. Interestingly, in this case almost all drugs, except L-Arginine, fit the pocket fully.

**Figure 5:**
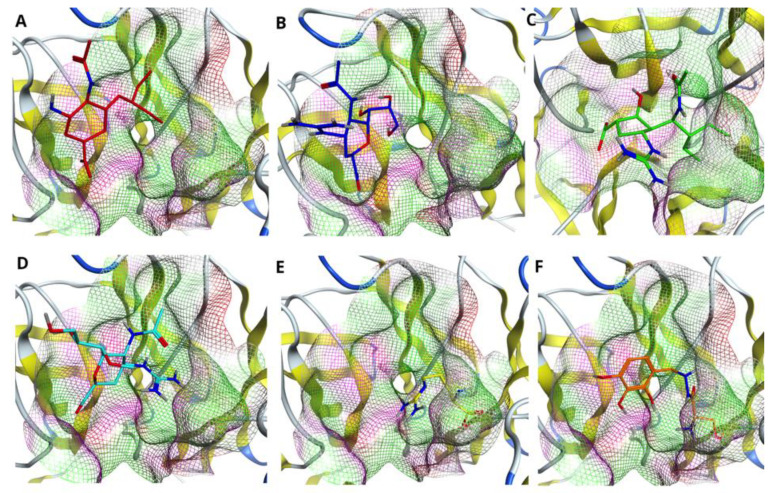
Comparing drug fit to double mutated NA binding pocket. Accommodation of the drugs (**A**) Oseltamivir (red), (**B**) Zanamivir (blue), (**C**) Peramivir (green), (**D**) Laninamivir (cyan), (**E**) L-Arginine (yellow) and (**F**) Benserazide hydrochloride (orange) in the pocket of H7N9 neuraminidase with double R294_119K mutation.

Next, we considered double R294_119K mutation inside the active site of N9 and fitness of the drugs to such a pocket. In this case, the mutation of two arginines at positions 294 and 119 into lysines not only affected the shape of the pocket, but also the positions of the drugs inside the pocket (see Figure 5A–F). In particular, all drugs, except Peramivir and Benserazide hydrochloride (see Figure 5C,F), are shifted with respect to the visual center of the pocket. Despite all these changes, Peramivir remains the drug that fits the pocket best in this case.

**Figure 6:**
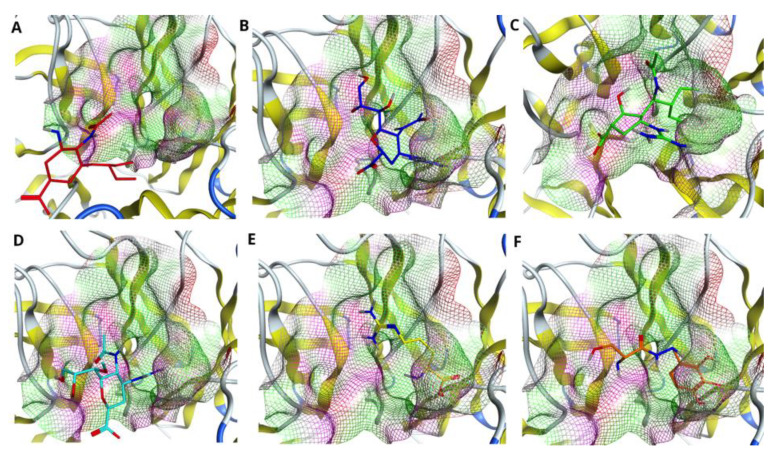
Comparing drug fit to triple mutated NA binding pocket. Accommodation of the drugs (**A**) Oseltamivir (red), (**B**) Zanamivir (blue), (**C**) Peramivir (green), (**D**) Laninamivir (cyan), (**E**) L-Arginine (yellow) and (**F**) Benserazide hydrochloride (orange) in the pocket of H7N9 neuraminidase with triple R294_119_372K mutation.

Finally, we considered the influence of triple R294_119_372K mutation on the binding poses and fitness of the drugs to the pocket of N9. As Figure 6 shows, the triple mutation dramatically affects the position of Oseltamivir inside the pocket (see Figure 6A). Less sensitive to such mutations are the drugs Laninamivir and L-Arginine; the position of the drug is shifted with respect to the visual center, however, not as significantly as in the case of Oseltamivir. The drugs Zanamivir, Peramivir and Benserazide hydrochloride are affected the least; they remain in the cavity of the enzyme. Although Benserazide hydrochloride scores the highest in this case (see Table 1, Table 2 and Table 3) and Zanamivir shows the smallest RMSD value amongst all drugs (see Table 6), it is worth noting that Peramivir is the only drug that interacts with three key residues at positions 119, 294 and 372 even after their mutation into lysines (please refer to Table 4, the last column).

### 2.6. Predicted Pharmacokinetic Properties for the Drugs

Table 7 reports the predicted absorption, distribution, metabolism, excretion and toxicity (ADMET) properties for Oseltamivir, Zanamivir, Peramivir, Laninamivir, L-Arginine and Benserazide hydrochloride according to the estimation by Douglas E.V. Pires et al. [15].

**Absorption:** As Table 7 shows, the best water solubility is found with Benserazide hydrochloride with a log mol/L value of −2.2, weaker solubility is found with the drug Oseltamivir (log mol/L −2.4) and the worst with drugs Zanamivir, Peramivir, Laninamivir and L-arginine with log mol/L values of −2.9. The high intestinal absorption is found with the drug Oseltamivir (74.5%), lower with the drugs L-arginine (34.5%) and Benserazide hydrochloride (36.2%) and the lowest with the drugs Zanamivir (21.2%), Peramivir (26.8%) and Laninamivir (27.4%). All drugs have relatively low skin permeability (please refer to the explanation provided by Douglas E. V. Pires et al. [15]).

**Distribution:** The distribution is characterized with the volume of distribution (VDss), fraction unbound (Fu) to the plasma proteins, blood–brain barrier (BBB) and central nervous system (CNS) permeability. According to Douglas E. V. Pires et al. [15], the higher the value of VDss, the more the drug is distributed in the tissue rather than in plasma. VDss is considered low if log VDss is < −0,15 and high if log VDss is > 0.45. In our case, all drugs show a quite low value of VDss, indicating their preference for blood plasma over tissue. From all studied compounds, the highest value of unbound fraction to the plasma proteins is found with Benserazide hydrochloride, lower with Oseltamivir and the lowest with the drugs Zanamivir, Peramivir, Laninamivir and L-arginine—as they show similar values.

All drugs show poor distribution to the brain with the log BB value close to the lowest threshold of -1. The same is related to the CNS permeability. All the compounds demonstrate the value of log PS < −3 (the lowest threshold), which indicates their poor capability to penetrate the CNS. However, as influenza is primarily a disease of the respiratory tissue, this does not prevent drug action in those most affected respiratory tissues such as lungs. 

**Metabolism:** The compound’s ability to inhibit cytochrome P450 can play a significant role in drug metabolism. In the current estimation, models of different isoforms of cytochrome P450, namely CYP1A2/CYP2C19/CYP2C9/CYP2D6/CYP3A4, were built based on the information collected from the 14,000 to 18,000 compounds capable of inhibiting cytochrome P450 [15]. The results show that our studied drugs are neither CYP2D6, CYP3A4 substrate, nor CYP1A2, CYP2C19, CYP2C9 inhibitors, which means that they do not hinder the metabolic process in the body.

**Excretion:** None of the considered drugs are organic cation transporter 2 (OCT2) substrate, which means they will not block the renal clearance process maintained through OCT2 [15].

**Toxicity:** All the drugs, except L-Arginine show no Ames toxicity, which means they are not sensitive to mutagenesis. The maximum tolerated dose (admissible threshold 0.477 log (mg/kg/day) is low for the drugs Zanamivir, Peramivir and L-Arginine and high for Oseltamivir, Laninamivir and Benserazide hydrochloride. The drugs show neither hepatotoxicity (except Benserazide hydrochloride) nor skin sensitization evidence. According to an interpretation by Douglas E.V. Pires et al. [15], this means that the compounds do not evoke liver-associated side effects and do not induce skin sensitization.

We give in addition in supplementary Table S1 data on pharmacological action considering a number of specific mutations in NA and the different drugs compared here including literature references. Moreover, further information on the reported ADMET properties of the drugs Peramivir and Zanamivir are listed in Table S2 based on the existing evidence. In summary, the ADMET properties compiled allow a direct comparison of the drugs in terms of tolerability and pharmacokinetics. All of them were purposely selected on the basis that they have already been used in treating severe influenza cases.

## 3. Discussion

It is well known that virus infection capabilities, in general, are very sensitive to protein mutations, and H7N9 avian type influenza virus is not an exception [16]. A mutation of R to K at position 294 in the binding site of N9 has already taken place, and led to Oseltamivir resistance [12]. Therefore, it is crucial to understand the influence of mutation in the active site as well as the mechanism of drug–enzyme interaction in order to avoid life-threatening accidents in the future. We introduced here all possible combination of mutations on three main residues—R294, R119 and R372—and assessed them systematically for the interaction of inhibitors in the enzyme binding pocket. In particular, R to K single, double and triple mutants were examined for their resistance and susceptibility to various antiviral drugs. The substitution of R to K in accordance with EDSSMat series is possible. The conservative mutation was examined in detail as one of the most probable mutation steps. The scoring of this matrix is achieved by distributing fewer penalties for non-aligned/disordered residues obtained through evolution [17]. Similarly, the Blosum matrix suggests that the replacement of R to K is highly favorable [14].

We used the molecular docking technique to structurally analyze the drug–enzyme interaction mechanism in wild type as well as mutated NAs. Results obtained from H-bond and salt bridge formation profile (please refer to Table 4 and Table 5), at first glance, brought us to the idea that the drugs Peramivir, Zanamivir and Laninamivir behave similarly. However, further analysis of the shapes of binding pockets upon mutations as well as drug fitness to them opened another perspective for evaluation. The residue substitutions, introduced in our study, changed the conformation of the pocket, which, in turn, affected the inhibitor–enzyme interaction, resulting in reduced binding capabilities for all drugs. Particularly influenced by mutations were the drugs Oseltamivir, L-Arginine and Benserazide hydrochloride, showing a huge shift with respect to the reference structure of the drug bound to the pocket of WT NA (see Table 6). The least affected by single mutations was the drug Peramivir with an RMSD value of less than 1 Å. Double and triple mutations had even higher impact on binding poses for all drugs, especially the one with R119_294K substitution. However, in this case, the drug Zanamivir was the one with the least displacement amongst the studied drugs (please refer to Table 6).

Mutation of the key residues in the active site of an enzyme reduces not only the affinity of the drug towards the pocket, but may also affect the normal activity of the enzyme itself. This is particularly well described for the influenza neuraminidase studied in the research work of Yen et al. [18].

However, in our case, the level of resistance triggered by the replaced residues when they come in contact with NA active site can be elucidated also in terms of reduction in H-bonds, notably in L-Arginine, where the residues Lys 119, Lys 294 and Lys 372 do not show any interactions. This makes an enzyme even more resistant to inhibitors, as these moieties are almost present in all other drug interactions with WT and mutant NAs. However, it is worth noting that R294 and Tyr 406 can be considered as the most significant residues for drug binding in the sense that they are involved in H-bond formations with almost all drug–enzyme complexes. In addition, we collected available evidence from direct pharmacological measurements on various mutated NA versions to prove the point that Peramivir and Zanamivir are effective against NA mutations as binding against a protein does not always translate into pharmacological activity (Table S1).

Moreover, keep in mind that of course always a specific mutation combination and regarding action and effectiveness in a clinical setting can only solidly be proven by clinical trials not attempted in this study. Instead, we consider our study rather an incentive and motivation for such clinical efforts.

Predicted pharmacokinetic properties (Table 7 and Table S2) did not show any toxicity or side effect for the considered drugs. They are well distributed in blood plasma, metabolized and excreted. In addition, there is no clinical trial evidence showing severe side effects for the above-mentioned drugs. In general, the drugs Oseltamivir, Zanamivir, Peramivir and Laninamivir are well tolerated but sometimes they trigger side effects such as mild nausea and vomiting [19,20,21,22,23,24]. As for the L-Arginine and Benserazide hydrochloride, there are no published data available. However, L-Arginine as an amino acid should be well tolerated by the human body.

The drugs Oseltamivir and Zanamivir are both commercially available [25]. However, Peramivir is available only in the USA, Korea, Japan and China [25], and Laninamivir only in Japan [25]. It is worth noting that the drugs Oseltamivir, Zanamivir, Peramivir and Laninamivir are regularly used during seasonal outbreaks of influenza virus [26,27]. In particular, Takemoto et al. [26] studied the clinical effectiveness of the above-mentioned NA inhibitors by treating 191 influenza-infected patients during the winter of 2012–2013 in Japan. The results revealed that Peramivir performed better in reducing fever as well as alleviating other influenza symptoms compared to Oseltamivir, Zanamivir and Laninamivir [26]. Similarly, Ishiguro and co-workers [27] studied the effect of these inhibitors in children with influenza types A and B. The study indicates the effectiveness of drugs in the case of treatment of influenza in elder children rather than in younger ones showing shorter recovery time in patients with type A virus compared to those with type B [27].

Independent experimental reports also underpin our modeling results, indicating that NA is more resistant to Oseltamivir than Peramivir, Zanamivir and Laninamivir [28]. He et al. [1] suggest that L-Arginine, Zanamivir and Benserazide hydrochloride may act as potential H7N9 NA inhibitors compared to Oseltamivir carboxylate for virus-carrying R294K mutation, and this strongly supports the data and coincides with our present investigation concerning other mutants with respect to drug binding affinity. Almost all H7N9 viruses show resistance to the inhibitor Oseltamivir, but the Suzhou strain does remain sensitive to the drug [29]; thus the strain-dependent sensitivity of the drug is a challenge.

After careful evaluation, we found that both Peramivir and Zanamivir may act best as a potential H7N9 viral NA inhibitor, as they show greater binding affinity and susceptibility towards NA compared to other drugs even in the case of mutation. Based on these results, we recommend to apply Peramivir in the case of single (R119K and R372K) mutations and Zanamivir in the case of double (R294_119K and R294_372K) mutations, as they both show high docking scores comparing three different methods as well as the lowest RMSDs regarding pocket fit with respect to the reference structure in these cases (please refer to Table 1, Table 2 and Table 3 and Table 6). However, in the case of triple (R294_119_372K) mutation, the application of both Peramivir and Zanamivir might achieve the best results. We came to this conclusion since, in the case of triple mutation, Peramivir is the only drug that formed hydrogen bonds with all three mutated residues of the binding pocket (see Table 4), demonstrating the strongest capabilities for the binding, while Zanamivir has shown the least RMSD compared to the reference structure, thus, showing its best fit to the active site. Therefore, combined treatment with Peramivir and Zanamivir will be an optimal solution in this case.

## 4. Main Limitations and Future Perspectives

Though compounds with known drug profiles are evaluated here in depth with the docking simulations for facing the future threat of different mutants with reference to H7N9 A influenza virus, which may instigate even a pandemic, the current study clearly has some limitations: The currently optimized binding affinity of the single, double and triple mutant complexes can only provide a first basis for guiding therapy and conducting future analysis in all possible manners, in particular experiments in vitro and in vivo (e.g., animal experiments).

We used three well established docking algorithms (AutoDock, Gold and MOE) and their overall scores are rather similar; however, they differed in details. Nevertheless, they unequivocally point to Peramivir and Zanamivir as the best approved drugs to treat patients also in the case of the most probable course of future mutation. Future mutations were tested only for the three most important positions and assuming the most likely similar substitution course to lysine. However, alternative amino acid substitutions are inherently less likely. To allow easy transfer and first recommendations for the clinic, we used only approved drugs and purposely did not look at further modifications. We are aware that such modifications may bind even better and hence we consider the approved drugs in this respect only as first leads for further development once a mutated strain becomes predominant.

We also strongly recommend exploring more synthetic analogues of Peramivir and Zanamivir as they represent near perfect leads for the mutants investigated in this study, allowing to cope with additional markers for drug resistance accompanying H7N9 influenza A virus.

Our findings did not touch upon other points important for virus transmission prevention such as developing a virus strain surveillance model, but we recommend, of course, as all health organizations concerned with flu, to monitor the progress of the influenza A viral strains in frequent intervals for better clinical studies and patient care.

## 5. Conclusions

The molecular docking technique has been applied to analyze the influence of mutation on drug resistance in the active site of NA from H7N9 influenza virus. The known anti-influenza drugs such as Oseltamivir, Zanamivir, Peramivir, Laninamivir, L-Arginine and Benserazide hydrochloride were analyzed for their binding capabilities against the pocket of wild type NA as well as the one with single, double and triple mutations in their key residues. According to the scores obtained from three independent docking protocols, we found that Peramivir and in some cases Zanamivir had the higher potency to bind NA enzymes compared to other studied drugs with the highest binding affinity towards wild type and single mutants and lower affinities toward double and triple mutants, respectively. Despite the fact that mutation changed the shape of the pocket and shifted the binding site for the studied drugs, bindings of Peramivir and Zanamivir were less affected by mutation. Moreover, Peramivir was the only drug that maintained interaction inside the pocket of triple N9 mutant with the key residues at positions 119, 294 and 372 even after their mutation to lysines, while Zanamivir was the drug with the lowest RMSD value (0.7 Å). Our findings suggest that Peramivir along with Zanamivir can be an optimal first available treatment solution in cases where H7N9 influenza virus acquires new drug-resistant mutations.

## 6. Computational Methods

### 6.1. Structure Derivation and Preparation

The 3D structures of N9 neuraminidases were obtained from protein data bank (PDB) under the following reference codes: 4MWQ [12] and 4MWW [12]. Mutations of R to K at positions 119 and 372 were performed with the help of the tools of Chimera software [30]. The 3D structures for Oseltamivir, Zanamivir, Peramivir, Laninamivir, L-Arginine and Benserazide hydrochloride were obtained from PubChem online drug databank under the following CIDs: 65028, 60855, 154234, 502272, 6322 and 26964, respectively. The structure preparation as well as energy minimization was achieved with the help of MOE (Molecular Operating Environment) software [31].

### 6.2. Molecular Docking

**AutoDock.** The AutoDock 4.2.6 [32] software along with MGL tools was obtained from the site of “The Scripps Research Institute” (http://autodock.scripps.edu/downloads/autodock-registration/autodock-4-2-download-page/ (accessed on 2 December 2021)) to perform molecular docking simulations.

The structures of proteins were protonated and the rotatable bonds for the ligands were clearly defined. The dimension of the grid box was set to 50 Å × 50 Å × 50 Å for all the docking simulations with spacing of 0.375 Å. The center of the grid box was placed so that it involved the residues of the active site of the protein and coincided with the center of ligand in the active site.

Lamarckian genetic algorithm (GA) was applied for all the docking simulations. The orientation, torsions and position of the drug molecule were set arbitrarily. 50 runs GA were performed for each docking and 2,500,000 energy evaluations were done with a total population size of 150. The final analysis of ligand conformations as well as their interaction profile with receptor was performed using PyMol software version 2.3 [33].

**GOLD**. Molecular docking using GOLD software was performed using version 5.5 [34]. Binding site residues were defined by specifying the crystal structure ligand coordinates bound to the protein and using the default cutoff radius of 6 Å, with the “detect cavity” option enabled. GOLD docking was carried out using the ChemPLP scoring function. For each drug molecule, 50 complexes were generated. The highest scoring drugs were selected as the most appropriate ones. 

**MOE**. Docking has been performed by selecting the default “Rigid Receptor” protocol. As a binding site, the coordinates of co-crystalized ligand atoms have been selected. The ligand placement was performed using the Triangle Matcher protocol. The top 30 poses were ranked by London dG scoring function and the resulting 5 poses were identified using Generalized-Born Volume Integral / Weighted Surface Area (GBVI/WSA dG) function. The conformations with more negative final score were considered as favorable.

RMSD values have been calculated using an online tool DockRMSD provided by Bell and Zhang [35].

### 6.3. Prediction of Pharmacokinetic Properties

The ADMET properties of the compounds were predicted with the help of pkCSM [15] online server tools available at (http://biosig.unimelb.edu.au/pkcsm/ (accessed on 4 September 2022)). The values were rounded to the first decimal.

### 6.4. PDB Files for the N9-Drug Complexes Obtained from Molecular Docking

The PDB files for all studied N9—drug complexes reported here were obtained from molecular docking simulations and can be accessed via the following link: https://www.biozentrum.uni-wuerzburg.de/bioinfo/computing/h7n9influenza/ (accessed on 4 September 2022).

In addition, they are provided as additional data in the supplement.

## Figures and Tables

**Table 1:** Lowest binding energies according to AutoDock software ^1^.

	R294 (4MWQ)	R294K (4MWW)	R119K	R372K	R294_119K	R294_372K	R119_372K	R294_119_372K
**Oselt.**	−8.3	−7.1	−7.0	−7.1	−6.2	**−6.9**	−6.4	**−6.2**
**Zan.**	−7.4	−7.4	−6.7	−6.6	−5.2	−6.4	−6.3	−5.2
**Per.**	**−8.7**	**−8.0**	**−7.2**	**−7.4**	**−6.3**	−6.2	**−6.6**	−6.0
**Lan.**	−7.7	**−8.1**	−6.7	−7.1	−5.0	−5.3	−6.5	−5.2
**L-Arg.**	−8.0	−7.1	−6.5	−6.4	−5.8	−6.4	**−6.6**	−6.3
**Bens.**	−7.1	−6.6	−6.3	−5.2	**−6.5**	−6.2	−6.0	**−6.6**

^1^ The best binding energies (kcal/mol) according to AutoDock docking simulations for the drugs Oseltamivir, Zanamivir, Peramivir, Laninamivir, L-Arginine and Benserazide hydrochloride with respect to the wild type as well as single, double and triple R to K mutations of N9 neuraminidase at positions 294, 119, 372. The top values among all studied drugs are shown in bold.

**Table 2:** ChemPLP scores according to GOLD software ^1^.

	R294 (4MWQ)	R294K (4MWW)	R119K	R372K	R294_119K	R294_372K	R119_372K	R294_119_372K
**Oselt.**	72.4	56.9	52.0	43.8	44.1	45.2	53.6	47.1
**Zan.**	89.3	75.2	51.7	**52.1**	**53.2**	**60.4**	52.3	45.0
**Per.**	**93.9**	**79.0**	**60.0**	50.0	**53.2**	49.1	**58.3**	46.8
**Lan.**	86.9	77.1	54.9	51.3	46.7	55.5	57.1	47.9
**L-Arg.**	46.8	45.3	41.5	34.8	41.0	41.5	42.0	40.0
**Bens.**	57.8	53.3	49.0	48.1	51.8	49.8	56.9	**55.5**

^1^ The highest values of ChemPLP score according to GOLD docking protocol for the drugs Oseltamivir, Zanamivir, Peramivir, Laninamivir, L-Arginine and Benserazide hydrochloride with respect to the wild type as well as single, double and triple R to K mutations of N9 neuraminidase at positions 294, 119, 372. The top scores are shown in bold.

**Table 3:** The S scores according to MOE software ^1^.

	R294 (4MWQ)	R294K (4MWW)	R119K	R372K	R294_119K	R294_372K	R119_372K	R294_119_372K
**Oselt.**	−6.0	−5.1	**−5.3**	−5.2	−4.9	−5.2	**−5.6**	−4.4
**Zan.**	−7.3	−6.4	−4.2	−4.7	**−5.6**	−5.0	**−5.6**	−5.0
**Per.**	**−7.7**	**−6.6**	**−5.3**	**−5.4**	**−5.6**	**−5.6**	−5.1	**−5.3**
**Lan.**	−6.6	−6.3	−4.7	−4.6	**−5.5**	−5.0	−4.9	−4.6
**L-Arg.**	−5.1	−5.0	−4.6	−4.4	−4.4	−4.4	−4.5	−4.7
**Bens.**	−5.1	−4.9	−5.0	−5.0	−5.2	−5.0	−5.4	**−5.4**

^1^ The top values of S score (kcal/mol) obtained from the docking simulations of MOE software for the drugs Oseltamivir, Zanamivir, Peramivir, Laninamivir, L-Arginine and Benserazide hydrochloride to the binding pocket of wild type as well as R to K mutations of N9 neuraminidase at positions 294, 119, 372. The top scores amongst all studied compounds are shown in bold.

**Table 4:** H- bond interaction profile for all the drugs compared ^1^.

	R294 (4MWQ)	R294K	R119K	R372K	R294_119K	R294_372K	R119_372K	R294_119_372K
**Oselt.**	Glu 120 **Arg 153** **Arg 372** **Arg 294**	**Arg 372** **Arg 119** **Arg 153**	Tyr 406 **Arg 372** **Arg 294**	**Arg 294** Tyr 406 **Arg 153**	**Arg 372** **Lys 294**	**Arg 153** **Arg 119**	**Lys 119** **Arg 153** **Arg 294**	**Lys 372**
**Zan.**	**Arg 372** **Arg 119** Asn 296 **Trp 180** Glu 229 **Asp 152**	**Tyr 406** **Arg 372** **Arg 119** **Lys 294** **Asp 152** **Trp 180**	**Trp 180** Arg 153 **Tyr 406** **Arg 294** **Arg 372**	**Trp 180** Arg 153 Glu 278 **Arg 294**	**Arg 372** **Lys 294** Glu 278	**Tyr 406** **Arg 119** **Asp 152** **Trp 180** Glu 278	Glu 278 **Arg 294** **Tyr 406** Glu 229 Arg 153 **Asp 152**	**Lys 119** Glu 120 **Asp 152** Glu 279 **Lys 372** **Tyr 406**
**Per.**	**Trp 180** Glu 229 **Arg 153** **Asp 152** Arg 119 **Arg 372** **Arg 294** **Tyr 406**	Arg 119 **Arg 372** **Asp 152** **Trp 180** Glu 229 **Arg 153**	**Trp 180** **Arg 153** **Arg 294** **Tyr 406** **Arg 372**	**Arg 153** **Arg 294** **Tyr 406**	**Asp 152** **Arg 372** **Lys 294** **Tyr 406**	**Lys 294** **Lys 372** **Tyr 406** **Asp 152**	Glu 229 **Trp 180** **Arg 153** **Tyr 406** **Arg 294**	Lys 119 **Lys 294** **Tyr 406** Glu 120 **Asp 152** **Lys 372**
**Lan.**	Arg 226 Glu 279 **Arg 372** Glu 229 **Arg 294** **Tyr 406** **Glu 278** Gly 349	Arg 119 **Arg 372** Asp 152 **Trp 180** **Arg 153** **Glu 278** **Lys 294**	**Trp 180** **Arg 153** **Glu 278** **Arg 294** **Arg 372** **Tyr 406**	Glu 278 **Arg 294** **Trp 180** **Arg 153**	**Arg 372** **Glu 278** **Lys 294**	**Glu 278** **Lys 294** Arg 119 **Arg 153** **Trp 180** Asp 152	**Tyr 406** **Arg 294** **Glu 278** **Arg 153** Glu 229 Asp 152	Lys 119 Glu 120 Lys 351 **Lys 372** **Tyr 406** Glu 427
**L-Arg.**	Arg 294 Arg 372 Glu 229 **Trp 180** **Asp 152**	**Trp 180** **Asp 152** **Tyr 406** Arg 372 Arg 119	**Trp 180** **Asp 152** Arg 372 **Tyr 406**	**Trp 180** Arg 153 **Tyr 406** Arg 294	**Tyr 406** Arg 153 **Asp 152** **Trp 180**	**Trp 180** **Asp 152** Arg 119	**Tyr 406** Glu 229 **Asp 152** Arg 153 **Trp 180** Lys 119	Glu 120 **Tyr 406** Glu 427
**Bens.**	**Trp 180** **Glu 229** **Asp 152** **Tyr 406** Arg 119 Arg 372	Arg 372 **Lys 294** **Tyr 406** **Trp 180** **Asp 152** **Glu 229**	**Trp 180** **Asp 152** Lys 119 Arg 372 **Tyr 406**	**Trp 180** Arg 153 **Tyr 406** **Arg 294**	**Tyr 406** **Lys 294** Arg 153 **Asp 152** **Trp 180**	Arg 153 **Trp 180** Glu 278 **Lys 294** **Tyr 406**	**Asp 152** **Trp 180** **Glu 229** **Tyr 406** **Arg 294**	Trp 180 Arg 153 Asp 152 **Tyr 406** Lys 294

^1^ Shown are the data for Oseltamivir, Zanamivir, Peramivir, Laninamivir, L-Arginine and Benserazide hydrochloride with respect to the wild type NAs as well as single, double and triple R to K mutants. The common residues from the catalytic domains of various NAs that are most frequently observed in H-bond formations with the drugs are highlighted in bold.

**Table 5:** Salt bridge interactions ^1^.

	R294 (4MWQ)	R294K (4MWW)	R119K	R372K	R294_119K	R294_372K	R119_372K	R294_119_372K
**Oselt.**	Arg 119 Arg 294 Arg 372	Arg 119 Arg 372	Arg 294 Arg 372	Arg 294 Lys 372	Lys 294 Arg 372	Arg 119 Lys 294 Lys 372	Arg 294	Lys 294 Lys 372
**Zan.**	Arg 119 Glu 120 Asp 152 Glu 229 Arg294 Arg 372	Arg 119 Arg 372 Glu 120 Asp 152 Glu 229	Glu 120 Asp152 Glu 229 Arg 294 Arg 372	Arg 119 Glu 120 Asp 152 Glu 229 Lys 372	Arg 372	Arg 119 Glu 120 Asp 152 Glu 229 Lys 372	Asp 152 Lys 372	Asp 152 Glu 279 Lys 294 Lys 372
**Per.**	Arg 119 Glu 120 Asp 152 Glu 229 Arg 294 Arg 372	Arg 119 Glu 120 Asp 152 Glu 229 Arg 372	Glu 120 Asp 152 Glu 229 Arg 294 Arg 372	Arg 119 Glu 120 Asp152 Glu 229 Arg 294 Lys 372	Glu 120 Asp152 Glu 279 Lys 294 Arg 372	Arg 119 Glu 120 Asp 152 Glu 279 Lys 372	Asp 152 Arg 294 Lys 372	Glu 120 Asp 152 Lys 294 Lys 372
**Lan.**	Arg 119 Glu 120 Asp 152 Glu 229 Arg 294 Arg 372	Arg 119 Arg 372 Glu 120 Asp 152 Glu 229	Arg 372 Glu 120 Asp 152 Glu 229	Arg 119 Glu 120 Asp 152 Glu 229 Lys 372	Glu 120 Asp 152 Arg 153 Glu 229 Glu 279	Arg 119 Glu 120 Asp 152 Glu 229 Lys 372	Glu 279 Arg 294 Lys 372	Asp 152 Glu 279 Lys 294 Lys 372
**L-Arg.**	Arg 119 Glu 120 Glu 229 Glu 279 Arg 294 Arg 372	Arg 119 Arg 372 Glu 120 Glu 279 Glu 229	Arg 294 Arg 372 Glu 120 Asp 152 Glu 229 Glu 279	Arg 119 Glu 120 Glu 229 Glu 279 Arg 294 Lys 372	Glu 120 Glu 229 Glu 279 Arg 372	Arg 119 Glu 120 Asp 152 Glu 229 Glu 279 Lys 372	Asp 152 Lys 372	Glu 120 Asp 152 Glu 229 Glu 279 Lys 372
**Bens.**	No Salt Bridge interactions	No Salt Bridge interactions	No Salt Bridge interactions	No Salt Bridge interactions	No Salt Bridge interactions	No Salt Bridge interactions	No Salt Bridge interactions	No Salt Bridge interactions

^1^ Salt bridge interaction profile for all the drugs (Oseltamivir, Zanamivir, Peramivir, Laninamivir, L-Arginine and Benserazide hydrochloride) with respect to the wild type NAs as well as single, double and triple R to K mutations.

**Table 6:** The RMSDs (Å) between the structures of the same drugs obtained from the docking to different pockets of N9 ^1^.

Mutant N9	Oseltamivir4MWQ	Zanamivir-4MWQ	Peramivir-4MWQ	Laninamivir-4MWQ	L-Arginine-4MWQ	Bens.-Hydrochl.-4MWQ
**R294K (4MWW)**	1.0	0.8	0.8	0.5	1.2	2.2
**R119K**	1.0	1.4	0.8	1.5	1.5	2.2
**R372K**	2.7	1.6	0.9	1.5	4.1	2.7
**R119_372K**	1.5	0.8	0.8	1.5	5.4	0.9
**R294_119K**	6.4	7.3	4.6	5.2	5.9	6.1
**R294_372K**	4.0	0.8	4.8	1.2	1.5	3.0
**R294_119_372K**	10.8	0.7	4.6	5.2	6.6	3.6

^1^ All the values are calculated with respect to the structure docked to the wild type NA (4MWQ) taken here as a reference.

**Table 7:** Predicted ADMET properties for the drugs Oseltamivir, Zanamivir, Peramivir, Laninamivir, L-Arginine and Benserazide hydrochloride.

Property	Model Name	Unit	Predicted Value					
			Oselt.	Zan.	Per.	Lan.	L-arg.	Bens.
Absorption	Water solubility	log mol/L	−2.4	−2.9	−2.9	−2.9	−2.9	−2.2
	Intestinal absorption (human)	% Absorbed	74.5	21.2	26.8	27.4	34.5	36.2
	Skin permeability	log Kp	−3.2	−2.7	−2.7	−2.7	−2.7	−2.7
	P-glycoprotein substrate	Yes/No	No	Yes	Yes	Yes	Yes	No
	P-glycoprotein I inhibitor	Yes/No	No	No	No	No	No	No
	P-glycoprotein II inhibitor	Yes/No	No	No	No	No	No	No
Distribution	VDss (human)	log L/kg	0.04	−0.08	−0.03	−0.2	−0.002	−0.2
	Fraction unbound (human)	Fu	0.6	0.4	0.4	0.4	0.4	0.9
	BBB permeability	log BB	−0.7	−1.2	−1.1	−1.2	−0.7	−1.5
	CNS permeability	log PS	−3.1	−4.7	−4.4	−4.4	−4.0	−4.8
Metabolism	CYP2D6 substrate	Yes/No	No	No	No	No	No	No
	CYP3A4 substrate	Yes/No	No	No	No	No	No	No
	CYP1A2 inhibitior	Yes/No	No	No	No	No	No	No
	CYP2C19 inhibitior	Yes/No	No	No	No	No	No	No
	CYP2C9 inhibitior	Yes/No	No	No	No	No	No	No
	CYP2D6 inhibitior	Yes/No	No	No	No	No	No	No
	CYP3A4 inhibitior	Yes/No	No	No	No	No	No	No
Excretion	Total Clearance	log ml/min/kg	0.9	0.3	−0.1	0.4	0.1	1.3
	Renal OCT2 substrate	Yes/No	No	No	No	No	No	No
Toxicity	Ames toxicity	Yes/No	No	No	No	No	Yes	No
	Max. tolerated dose (human)	log mg/kg/day	0.5	0.4	0.4	0.5	0.4	0.6
	hERG I inhibitor	Yes/No	No	No	No	No	No	No
	hERG II inhibitor	Yes/No	No	No	No	No	No	No
	Oral Rat Acute Toxicity (LD50)	mol/kg	2.7	2.5	2.5	2.5	2.5	2.2
	Oral Rat Chronic Toxicity	log mg/kg_bw/day	1.1	2.7	2.9	2.5	2.2	3.7
	Hepatotoxicity	Yes/No	No	No	No	No	No	Yes
	Skin Sensitization	Yes/No	No	No	No	No	No	No
	T. Pyriformis toxicity	log ug/L	0.1	0.3	0.3	0.3	0.3	0.3
	Minnow toxicity	log mM	2.3	5.8	3.0	5.3	3.3	3.9

## Data Availability

All data are fully available and contained in the manuscript and its supplementary materials.

## Supplementary Materials

The following supporting information can be downloaded at: https://www.mdpi.com/article/10.3390/molecules27185920/s1, Table S1: Pharmacological action against NA mutations by Peramivir and Zanamivir; Table S2: ADMET properties of drugs Peramivir and Zanamivir. References [36,37,38,39,40,41,42,43,44,45,46,47,48,49,50,51,52,53,54,55,56,57,58] are cited in the supplementary materials.

## Sample Availability

All data are made available here. These are in silico data, no chemical samples.
