# Supplementary material for: In Silico Studies Reveal Peramivir and Zanamivir as an Optimal Drug Treatment Even If H7N9 Avian Type Influenza Virus Acquires Further Resistance
Source: Molecules. 2022 Sep 12;27(18):5920. doi: 10.3390/molecules27185920 (PMC9503969; doi:10.3390/molecules27185920)
Supplement: Supplementary file 1 [file molecules-27-05920-s001.zip › molecules-1876953-supplementary.pdf]

Supplementary Material

In Silico Studies Reveal Peramivir and Zanamivir as an Optimal Drug Treatment Even if H7N9 Avian Type Influenza Virus Acquires Further Resistance

Table S1. Pharmacological action against NA mutations by Peramivir and Zanamivir [1].

<table>
<tr><th>Drug
(Mode of administration)</th><th>Mutations reported in NA gene</th><th>Performance of the drug against the mutation</th><th>Experimental models used for NA-Inhibitors (anti-viral) testing</th></tr>
<tr><td>Peramivir (Intravenous administration)</td><td rowspan="2">Arg 292 Lys [10]
Arg 294 Lys [1]
Arg 289 Lys [43]
Glu 119 Val [39]
His 274 Tyr [42]
Iso 222 Lys [42]
Iso 222 Arg [46]
Iso 222 Val [48]
Iso 222 Lys [46]
Gly 146 Arg [47]
Gln 136 Lys [49]
Asn 295 Ser [50]
Val 116 Ala [51]
His 117 Val [47]</td><td>Acts on the NA active site where the drug interacts with sialic acid causing distortion of pyranose ring by enabling hydrolytic cleavage [33]. The side chain groups (C4 guanidino & pentyl ether) present in the drug promotes higher binding affinity to the enzyme (NA) active site [34, 35]. There by hindering the entry of corresponding viruses and their release</td><td rowspan="2">Animal Models [47]
Mice
Ferrets
Golden Syrian Hamsters
Guinea pigs
Cotton rats
Chickens
Non-Human Primates
Horses

Cells lines utilized [55]
HEK 293T
MDCK (Madin-Darby canine kidney epithelial cells)
MDCK-SIAT1</td></tr>
<tr><td>Zanamivir (Inhalation & Intravenous administration)</td><td>Substitution of 4-guanidino in place of C4-OH yields zanamivir and is based on the analog DANA. Enhanced binding of 10,000-fold is reported with zanamivir. The drug has higher binding affinity towards the NA catalytic site and thereby blocking the interaction between the sialic acid and NA [37, 44]. Hence overall alteration of viral assembly and release is triggered by the drug binding, as a result, further viral release from the host cell and infection is therefore prevented</td></tr>
</table>

[1]The table above represents the previously reported mutations and the performance of drugs Peramivir and Zanamivir recommended in this study against such NA mutations. The animal and cell line models used in the anti-viral screening of NA-inhibitors are also listed. The sensitivity of H7N9 to peramivir has also been briefly reported in the in-vitro study conducted by Cao R-Y et al where they have utilized MDCK cells which are highly susceptible to H7N9 strains [36].

Table S2. ADMET properties of drugs Peramivir and Zanamivir. [1.]

Drugs of Interest	Pharmacokinetic Properties reported	Adverse effects	References
Peramivir	Less binding to human plasma protein (<30%)	>10% Diarrhea	[37, 38, 39, 54]
	Effective renal clearance (~90%) without interfering with multiple metabolic pathways in humans		
	No carcinogenicity		
	No immunotoxicity		
	No cytotoxicity		
	No mutagenicity		
	No predicted hERG inhibition		
	Not an inhibitor of CYP1A2, CYP2C19, CYP2C9, CYP2D6 & CYP3A4		
	Low GI absorption and no BBB permeant		
	Higher penetration of the drug to lower airways		
Zanamivir	No reported hepatotoxicity	1-10% rash, diarrhea, liver injury, neutro-penia, renal failure & increase in the levels of liver enzymes (AST, ALT).	[42,52,53,54]
	No reported skin sensitization		
	Not an inhibitor of hERG I & hERG II		
	No detected AMES toxicity		
	Not an inhibitor of CYP1A2, CYP2C19, CYP2C9, CYP2D6, CYP3A4		
	Not an inhibitor of P-glycoprotein I & II		
	Acts as a substrate of P-glycoprotein, CYP2D6 & CYP3A4		
	Not a renal OCT2 substrate		
	Inhibitor of human sialidases NEU2 & NEU3		
	Less binding to human plasma protein (<10%)		
	Non-Carcinogenic		
	Readily biodegradable		

[1] The reported ADMET properties of the drugs Peramivir and Zanamivir are listed based on the existing evidence. They are in correlation with the predicted pharmacokinetic properties that are previously listed in the table 7.

33. Mtambo, S.E.; Amoako, D.G.; Somboro, A.M.; Agoni, C.; Lawal, M.M.; Gumede, N.S.; Khan, R.B.; Kumalo, H.M. Influenza Viruses: Harnessing the Crucial Role of the M2 Ion-Channel and Neuraminidase toward Inhibitor Design. *Molecules* 2021, *26*, doi:10.3390/molecules26040880.
34. Mclaughlin, M.M.; Skoglund, E.W.; Ison, M.G. Peramivir: An Intravenous Neuraminidase Inhibitor. *Expert Opin Pharmacother* 2015, *16*, 1889–1900, doi:10.1517/14656566.2015.1066336.
35. Scott, L.J. Peramivir: A Review in Uncomplicated Influenza. *Drugs* 2018, *78*, 1363–1370.
36. Cao, R.Y.; Xiao, J.H.; Cao, B.; Li, S.; Kumaki, Y.; Zhong, W. Inhibition of Novel Reassortant Avian Influenza H7N9 Virus Infection in Vitro with Three Antiviral Drugs, Oseltamivir, Peramivir and Favipiravir. *Antivir Chem Chemother* 2014, *23*, 237–240, doi:10.3851/IMP2672.
37. CHMP *IV Zanamivir, INN Zavamivir*; EMA/498672/2011. (www.ema.europa.eu)
38. Mtambo, S.E.; Kumalo, H.M. In Silico Drug Repurposing of FDA-Approved Drugs Highlighting Promacta as a Potential Inhibitor of H7N9 Influenza Virus. *Molecules* 2022, *27*, 4515, doi:10.3390/molecules27144515.
39. Mtambo, S.E.; Ugbaja, S.C.; Mushebenge, A.G.; Abubakar, B.H.; Ntuli, M.L.; Kumalo, H.M. Intermolecular Mechanism and Dynamic Investigation of Avian Influenza H7N9 Virus' Susceptibility to E119V-Substituted Peramivir–Neuraminidase Complex. *Molecules* 2022, *27*, doi:10.3390/molecules27051640.
40. Funatsu, Y.; Tasaka, S.; Asami, T.; Namkoong, H.; Fujiwara, H.; Iketani, O.; Yagi, K.; Kimizuka, Y.; Ishii, M.; Nishimura, T.; et al. Pharmacokinetics of Intravenous Peramivir in the Airway Epithelial Lining Fluid of Healthy Volunteers. *Antivir Ther* 2016, *21*, 621–625, doi:10.3851/IMP3096.
41. Wu, X.; Xiao, L.; Li, L. Research Progress on Human Infection with Avian Influenza H7N9. *Front Med* 2020, *14*, 8–20.
42. Marjuki, H.; Mishin, V.P.; Chesnokov, A.P.; de La Cruz, J.A.; Davis, C.T.; Villanueva, J.M.; Fry, A.M.; Gubareva, L. v. Neuraminidase Mutations Conferring Resistance to Oseltamivir in Influenza A(H7N9) Viruses. *J Virol* 2015, *89*, 5419–5426, doi:10.1128/jvi.03513-14.
43. Tran-To Su, C.; Ouyang, X.; Zheng, J.; Kwoh, C.K. Structural Analysis of the Novel Influenza A (H7N9) Viral Neuraminidase Interactions with Current Approved Neuraminidase Inhibitors Oseltamivir, Zanamivir, and Peramivir in the Presence of Mutation R289K. *BMC Bioinformatics* 2013, *14*, doi:10.1186/1471-2105-14-S16-S7.
44. Fleming, D.M. Zanamivir in the Treatment of Influenza. *Expert Opin Pharmacother* 2003, *4*, 799–805.
45. Burranboina, K.K.; Kumar, K.M.; Manjunatha Reddy, G.B.; Yogisharadhya, R.; Prashantha, C.N.; Dhulappa, A. GC-MS Analysis, Molecular Docking and Pharmacokinetic Studies of Various Bioactive Compounds from Methanolic Leaf Extracts of Leucas Aspera (L) against Anti - Capripox Viral Activity. *Chemical Data Collections* 2022, *39*, doi:10.1016/j.cdc.2022.100873.
46. Marjuki, H.; Mishin, V.P.; Chesnokov, A.P.; Jones, J.; de La Cruz, J.A.; Sleeman, K.; Tamura, D.; Nguyen, H.T.; Wu, H.S.; Chang, F.Y.; et al. Characterization of Drug-Resistant Influenza a(H7N9) Variants Isolated from an Oseltamivir-Treated Patient in Taiwan. *Journal of Infectious Diseases* 2015, *211*, 249–257, doi:10.1093/infdis/jiu447.

47. Caceres, C.J.; Seibert, B.; Cargnin Faccin, F.; Cardenas-Garcia, S.; Rajao, D.S.; Perez, D.R. Influenza Antivirals and Animal Models. *FEBS Open Bio* 2022, *12*, 1142–1165.
48. Simon, P.; Holder, B.P.; Bouhy, X.; Abed, Y.; Beauchemin, C.A.A.; Boivin, G. The I222V Neuraminidase Mutation Has a Compensatory Role in Replication of an Oseltamivir-Resistant Influenza Virus A/H3N2 E119V Mutant. *J Clin Microbiol* 2011, *49*, 715–717, doi:10.1128/JCM.01732-10.
49. Hurt, A.C.; Holien, J.K.; Parker, M.; Kelso, A.; Barr, I.G. Zanamivir-Resistant Influenza Viruses with a Novel Neuraminidase Mutation. *J Virol* 2009, *83*, 10366–10373, doi:10.1128/jvi.01200-09.
50. Earhart, K.C.; Elsayed, N.M.; Saad, M.D.; Gubareva, L. v.; Nayel, A.; Deyde, V.M.; Abdelsattar, A.; Abdelghani, A.S.; Boynton, B.R.; Mansour, M.M.; et al. Oseltamivir Resistance Mutation N294S in Human Influenza A(H5N1) Virus in Egypt. *J Infect Public Health* 2009, *2*, 74–80, doi:10.1016/j.jiph.2009.04.004.
51. Boltz, D.A.; Douangngeun, B.; Phommachanh, P.; Sinthasak, S.; Mondry, R.; Obert, C.; Seiler, P.; Keating, R.; Suzuki, Y.; Hiramatsu, H.; et al. Emergence of H5N1 Avian Influenza Viruses with Reduced Sensitivity to Neuraminidase Inhibitors and Novel Reassortants in Lao People's Democratic Republic. *Journal of General Virology* 2010, *91*, 949–959, doi:10.1099/vir.0.017459-0.
52. Oakley, A.J.; Barrett, S.; Peat, T.S.; Newman, J.; Streltsov, V.A.; Waddington, L.; Saito, T.; Tashiro, M.; McKimm-Breschkin, J.L. Structural and Functional Basis of Resistance to Neuraminidase Inhibitors of Influenza B Viruses. *J Med Chem* 2010, *53*, 6421–6431, doi:10.1021/jm100621s.
53. Eiland, L.S.; Eiland, E.H. *Zanamivir for the Prevention of Infl Uenza in Adults and Children Age 5 Years and Older*; 2007; Vol. 3;.
54. Świerczyńska, M.; Mirowska-Guzel, D.M.; Pindelska, E. Antiviral Drugs in Influenza. *Int J Environ Res Public Health* 2022, *19*.
55. Baz, M.; Abed, Y.; Boivin, G. Characterization of Drug-Resistant Recombinant Influenza A/H1N1 Viruses Selected in Vitro with Peramivir and Zanamivir. *Antiviral Res* 2007, *74*, 159–162, doi:10.1016/j.antiviral.2006.10.012.
